# Dysregulation of miR-155 Expression in Professional Mixed Martial Arts (MMA) Fighters

**DOI:** 10.7759/cureus.34944

**Published:** 2023-02-13

**Authors:** Dominick Cabrera, Kayla Thompson, Julius D Thomas, Corey Peacock, Jose Antonio, Jaime L Tartar, Aurelien Tartar

**Affiliations:** 1 Psychology, Nova Southeastern University Dr. Kiran C. Patel College of Osteopathic Medicine, Davie, USA; 2 Psychology, Nova Southeastern University, Davie, USA; 3 College of Health Care Sciences, Nova Southeastern University, Davie, USA; 4 Biological Sciences, Nova Southeastern University, Davie, USA

**Keywords:** neuro inflammation, mirna signature, athlete, gene expression profile, traumatic brain injury

## Abstract

Psychological and physical stress can induce dysregulation of gene expression via changes in DNA methylation and microRNA (miRNA) expression. Such epigenetic modifications are yet to be investigated in professional Mixed Martial Arts (MMA) fighters subject to highly stressful training involving repetitive head impacts. This study examined differences in DNA methylation and miRNA expression in elite MMA fighters compared to active controls. Global methylation differences between groups were assessed via a LINE-1 assay. At the same time, PCR arrays were used to estimate differential expression in samples of 21 fighters and 15 controls for 192 different miRNAs associated with inflammatory diseases. An Independent-Samples *t*-Test found no significant difference in LINE-1 methylation between groups. However, an Independent-Samples Mann-Whitney U Test revealed a significant upregulation in the expression of miR-155 in MMA fighter plasma. Since miR-155 has been recognized as an important regulator of neuroinflammation, this dysregulation suggests a possible epigenetic mechanism responsible for chronic inflammation associated with professional-level MMA training. Consistent with other published works, this study highlights the potential of miR-155 not only as a biomarker for monitoring long-term health risks linked to head trauma but also as a target to remediate the impact of chronic neuroinflammation.

## Introduction

Traumatic brain injury (TBI) often involves blunt force impacts to the head, causing rapid percussive or rotational damage to the brain [1]. This can result in bleeding, brain cell loss, and stretching of axons, often producing cognitive impairment, neuropsychiatric symptoms, dementia, and parkinsonism. Mild traumatic brain injury (mTBI) accounts for about 85% of total cases worldwide, posing a major concern for healthcare providers tasked with diagnosing and treating patients suffering the various repercussions. mTBI is often undiagnosed, especially among contact sports athletes motivated to persist in competition and refuse to disclose their symptoms [1]. The impact of both TBI and mTBI on athletes has recently been highlighted by highly publicized cases of chronic traumatic encephalopathy (CTE) [2]. Many factors, including psychological stress, may contribute to the progressive tauopathy seen in CTE; however, it is still uncertain how frequency and severity of injury factor into the time course of neuropsychological decline. While recent diagnoses have been made in athletes in their 20s, CTE has most frequently been observed in the brains of older retired athletes since it can only be currently confirmed by observing the brains of deceased athletes. A recent study diagnosed CTE in 177 of the 202 (87%) deceased American football players studied [3]. The severity of exposure to repetitive head impacts (RHI) necessary to place one at risk for CTE remains unclear. However, research supports that long-term inflammation mediated by microglial activation and tau pathology contributes significantly to disease progression and is likely associated with more prolonged exposure to repetitive head trauma [4]. Experimental research also supports the idea that this chronic neuroinflammation causes progressive neurodegeneration, which can potentially be treated long after the instance of TBI [5].

Noninvasive diagnosis of CTE remains problematic. Some current efforts have focused on dual and concomitant objectives: (i) explaining the underlying molecular mechanisms of diseases resulting from RHI and (ii) identifying molecular markers of disease progression [1,4,6,7]. For example, recent research has suggested persistent changes in DNA methylation with TBI. Exposure to RHI or an acute bout of exercise can rapidly change DNA methylation levels in associated tissues [8,9]. This epigenetic dysregulation could result in various diseases depending on which gene regions are differentially methylated, such as those associated with limbic system structures and Brain-Derived Neurotrophic Factor (BDNF) secretion [9].

Similarly, miRNA microarray and real-time qPCR (RT-qPCR) techniques have both been used to demonstrate the differential expression of microRNA (miRNAs) associated with the cerebral cortex (e.g., miR-21) following TBI in a rat model [10,11]. Various miRNAs circulating in plasma showed altered expression patterns soon after TBI in mice [12] and humans [13]. For instance, miR-155 is involved in the upregulation of microglia-mediated neuroimmune response, implicating its inhibition as a therapeutic method [14]. The overexpression of miR-155 has increased dendritic cell apoptosis and enhanced IL-12p70 production, which plays a role in Interferon-gamma (IFN-γ)-producing T cell development and natural killer cell activation [15]. More generally, the effectiveness of miRNAs as diagnostic markers for a wide range of diseases [16-19] or injuries [20] is a rapidly expanding area of research.

Based on these findings, the current study evaluated differences in DNA methylation and expression of miRNAs associated with chronic inflammation and various cancers between professional MMA fighters and a control group of non-contact sports athletes. In contrast to previous studies [1], the fighters tested were active, elite professionals that competed in the Ultimate Fighting Championship (UFC) or Bellator. They tend to experience RHI and are at risk for diseases associated with chronic brain inflammation [1]. While research into CTE has typically been observed in older retired athletes, little is known of epigenetic modification in younger elite professionals currently active in their careers. This may contribute to the risk of sustained neuroinflammatory disease experienced later in life.

Part of the data presented in this article was previously presented as a meeting abstract at the 2019 International Society of Sports Nutrition (ISSN) Annual Scientific Meeting in June 2019, at the 2019 Society for Neurosports Annual Scientific Meeting in November 2019, and at the 2022 International Behavioral Neuroscience Society (IBNS) Annual Scientific Meeting in June 2022.

## Materials and methods

Participants

As part of a larger study investigating the effects of contact sport participation on inflammatory biomarkers and neurobehavioral performance, 21 male MMA fighters training for professional combat and 15 male age-matched professional athlete controls not experiencing RHI provided data for this analysis. Athletes were tested at the Nova Southeastern University (NSU) Health Profession's Annex in the Fight Science laboratory. All participants underwent an informed consent process following the Declaration of Helsinki and an approved IRB protocol submitted to the institutional review board of Nova Southeastern University (approval number: 2018-08-14).

Body composition

Body composition was assessed with a dual-energy X-ray absorptiometry machine (DXA) (Model: Hologic Horizon W; Hologic Inc., Danbury CT, USA). Quality control calibration procedures were performed on a spine phantom. Subjects were instructed to come to the laboratory after at least a 3-hour fast and no prior exercise. Subjects wore typical athletic clothing and removed metal jewelry. The research participants were positioned supine on the DXA within the borders delineated by the scanning table. Each whole-body scan took approximately seven minutes.

DNA and RNA extractions

Blood samples (3 mL) were collected from participants and centrifuged to separate plasma. Saliva samples (1 mL) were also collected via passive drool. DNA was extracted from saliva samples using the QIAamp DNA Investigator kit per manufacturer protocol. In contrast, RNA samples were extracted from the plasma samples using the miRNeasy Serum/Plasma Kit (both kits from QIAGEN, Valencia, CA, USA). All extractions were performed in a QIAcube instrument, and sample purity and concentration assessments were measured using a Nanodrop Lite Spectrophotometer (ThermoFisher Scientific, Waltham, Massachusetts, USA).

DNA methylation analysis

Global methylation differences between groups were assessed via a LINE-1 assay (surrogate global DNA analysis), a product of Active Motif (Carlsbad, CA, USA), according to the assay protocol. 1 µL of each DNA sample was prepared for the LINE-1 methylation assay via a Msel digestion. Approximately 100 ng of digested sample DNA was used per well, and samples were run in triplicate.

miRNA expression analysis

Each extracted RNA sample was converted to cDNA using the miScript II RT Kit (QIAGEN). 1 µL of each athlete's cDNA was combined into a pool with 10x of RNAse-free water. The Human Inflammatory Response & Autoimmunity and Human Serum & Plasma miScript miRNA PCR Arrays (QIAGEN) were used to estimate differential expression between MMA fighters and the control group for 192 miRNAs. Pooled samples (2 µL) were used as templates for each reaction, following the manufacturer's protocol. RT-qPCR was then used to quantify the expression of miR-155 in individuals of both groups by combining cDNA samples with SNORD61 and miR-155 primers, respectively, using miScript Primer Assays (QIAGEN). Dilutions (1:10) of all individual cDNA samples were used for these assays, and reactions were run in duplicates. The relative expression ratio for miR-155 in MMA fighters vs. matched controls was determined from quantitative PCR results to the control miRNA, SNORD61, and performed in the ROCHE LC96 Application Software [21].

Statistical analyses

Age and body composition differences were determined via paired samples t-tests. The effect of group status on outcome measures was analyzed via Independent Samples t-tests. In the case of non-normal distribution, where the normality assumption was not met, an Independent-Samples Mann-Whitney U Test was carried out. All calculations were conducted using an SPSS statistical package (version 26, IBM, Armonk, NY, USA). All reported p-values are two-tailed with an a priori significance level of p <0.05.

## Results

**Figure 1:**
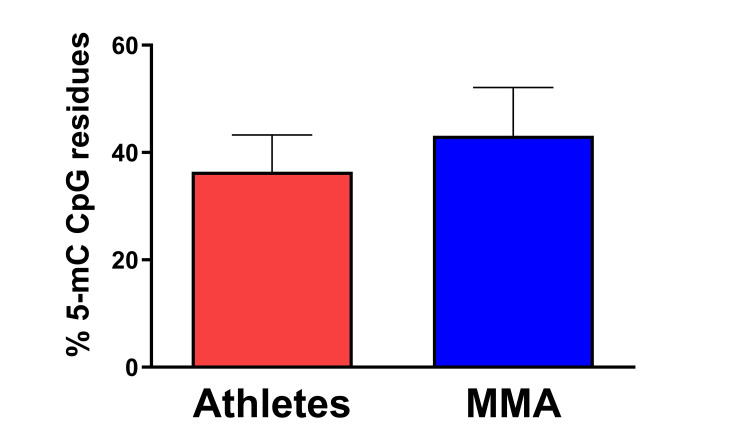
Comparative analysis of DNA methylation levels in saliva samples collected from non-contact athletes and Mixed Martial Arts (MMA) fighters. Although lower DNA methylation levels characterized Mixed Martial Arts (MMA) fighters, no significant difference between groups was found. Error bars represent the standard error of the mean. MMA = Mixed Martial Arts, 5mC = 5-methylcytosine, CpG = 5’-cytosine-phosphodiester bond-guanine-3’.

The sample sizes and respective body composition parameters are summarized in Table 1. The relatively small size of both groups is largely due to the challenges associated with identifying and recruiting individuals who met the stringent criteria for study enrollment (active and elite professional male athletes). Sample sizes, or apparent differences between both groups, did not impact the results described below. The statistical tests used for data interpretation were specifically selected to account for these conditions. As expected, both groups showed no significant difference in age, although there were variations in parameters associated with bone and lean mass composition (Table 1). High-quality DNA and RNA samples were obtained for all subjects. DNA methylation analysis suggested that global DNA methylation levels, measured using Long Interspersed Nucleotide Element 1 (LINE-1) repeats, comprise approximately 17% of the total genome and act as a surrogate estimate for global DNA methylation levels, were similar between the two groups. As illustrated in Figure 1, an independent samples t-test found no significant difference between groups in LINE-1 methylation t(34) = 0.60, p = 0.55, d = 0.20. In contrast, the miRNA PCR arrays indicated 28 miRNAs with differential expression between the pooled samples of MMA fighters and control athletes (Table 2). These miRNA dysregulations indicate that, as suggested by previous studies, unique molecular signatures may be detected in MMA fighters and are potentially related to the high frequency of RHI experienced by professional fighters relative to other athletes [1].

**Table 1:** Age and body composition measures for the Mixed Martial Arts (MMA) fighters and athlete controls. Both groups were 100% males. All measures are indicated as mean ± standard deviations. Statistical analyses correspond to independent samples t-tests. Asterisks indicate p < 0.05. MMA = Mixed Martial Arts, BMC = Bone Mineral Content, BMD = Bone Mineral Density. ^#^Due to height restrictions of the DXA, one participant in the MMA group (height = 213 cm) was unable to be included in analyses of BMC, BMD, Body fat, and Lean mass

	MMA (N=21^#^)	Athletes (N=15)	t value	p-value
Age	29.8±5.5	35.1±11.2	1.89	0.07
Height (cm)	183.9±10.6	176.8±8.0	2.18	0.04*
BMC (kg)	3.8±0.6	3.1±0.6	3.47	<0.01*
BMD (g/cm^2^)	1.5±0.1	1.3±0.1	3.79	<0.01*
Body Fat (%)	21.6±17.8	17.7±9.0	0.83	0.41
Lean Mass (kg)	70.5±11.7	62.6±7.2	2.20	0.03*

**Table 2:** Differentially expressed miRNAs in pooled plasma samples of Mixed Martial Arts (MMA) fighters vs. age-matched athlete controls.

Array	Differentially Expressed miRNAs Observed from Microarray with Pooled Samples
Human Serum and Plasma	miR-1; miR-143; miR-148a: miR-7: miR-200c: miR-10b: miR-155: miR-193a: miR-296: miR-211: miR-192: miR-10a: miR-196a: miR-128; miR-134
Human Inflammatory Response & Autoimmunity	miR-424; miR-130b; miR-30c; miR-449a; miR-130a; miR-181d; miR-29b; miR-145; miR-202; miR-374a; miR-17; miR-125a; miR-15a

**Figure 2:**
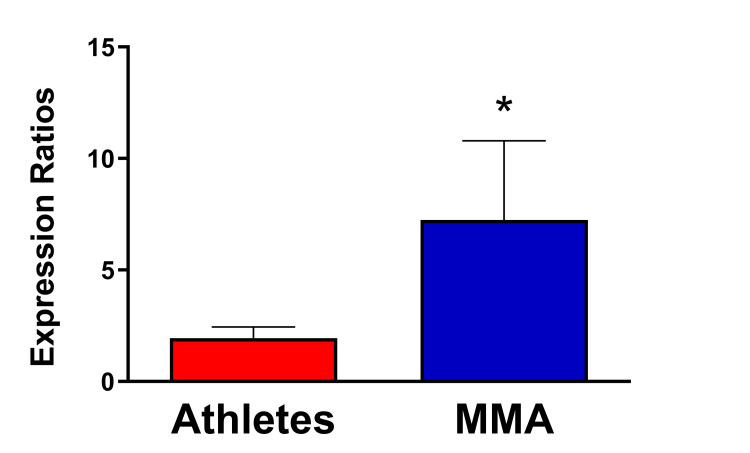
Comparative analysis of miR-155 expression levels in plasma samples collected from non-contact athletes and Mixed Martial Arts (MMA) fighters. A Mann-Whitney U test found miR-155 significantly overexpressed in the group of MMA fighters, indicating that the stress of professional MMA training may predispose fighters to display unique and potentially diagnostic molecular patterns. Error bars represent the standard error of the mean. Asterisk represents p < 0.05. MMA = Mixed Martial Arts.

To further validate the preliminary miRNA expression dysregulation data, qPCR-based analyses were performed to measure differential expression in individual samples. These analyses used SNORD61 and miR-155 gene-specific primers. They assessed the expression of miR-155 relative to SNORD61, which was used as a reference with expression levels assumed to be constant in all conditions (control athletes and MMA fighters). As illustrated in Figure 2, miR-155 was found to be more expressed than SNORD61, and a Mann-Whitney U test found significantly higher expression of miR-155 in the group of MMA fighters (mean: 13.64, n = 11) compared to the group of active controls (mean: 8.10, n = 10) (U = 26.00, P = 0.043). This result confirms the high-throughput, pooled sample assays. The other miRNAs listed in Table 2 may also represent individual targets for further analyses of dysregulated gene expression patterns in MMA fighters.

## Discussion

The current study sought to determine if professional MMA fighters experience dysregulated molecular patterns at the DNA or RNA levels. In the time following a typical sparring practice - which occurs twice a week for months leading up to a fight - these athletes likely experience molecular changes in measures related to muscle and bone synthesis, angiogenesis, and pro- and anti-inflammatory systems. While some of these changes may be part of beneficial adaptations to exercise, prolonged exposure to dysregulation of some of these measures may underlie tauopathy or other neuroinflammatory states observed in CTE, suggesting that altered molecular patterns may be mined for potential biomarkers associated with disease risk or progression.

Data generated throughout this study suggest that miRNA expression patterns have greater potential as a biomarker of neural integrity than DNA methylation. No significant difference in LINE-1 methylation between the two groups indicates that, despite the stress of MMA training, these fighters are not at any greater risk of developing dysregulation of global methylation (Figure 1). More precise measurements of DNA methylation at specific gene regions may reveal differences in MMA fighters relative to an athlete control group and support the growing body of literature linking epigenetic modifications with TBI [9,22] - including TBI experienced in contact sport competition [23]. While DNA methylation appeared stable among experimental groups, differential expression of select miRNAs was readily identified between MMA fighters and the matched athlete's control group (Table 2). These results not only serve to confirm a previously published study that highlighted changes in miRNA gene expression in amateur MMA fighters [1], but they are also consistent with previous efforts that assessed differential miRNA expression after TBI [13,24]. Several of the miRNAs found to be dysregulated in the present study, including miR-128, miR-15a, and miR-10b, were also differentially expressed in brain tissue samples from individuals diagnosed with CTE or Amyotrophic Lateral Sclerosis (ALS) [24].

Changes in miRNA expression following TBI have also been detected in plasma [13] and have implicated miR-155, especially in rodent models, where miR-155 levels increased after TBI (reviewed in [25]). In agreement with these observations, miR-155 up-regulation in serum has recently been associated with the number of hits to the heads (HTH) experienced by amateur MMA fighters [1]. Herein, a significant difference in the expression of miR-155 was measured (Figure 2) in professional fighters relative to controls, suggesting that these athletes may experience chronic overexpression of this miRNA and, therefore, may be at a greater risk for experiencing chronic inflammation or developing diseases such as CTE or ALS as a result of the stress and intensity associated with their training. The critical role of miR-155 as a regulator of neuroinflammation has been recently reviewed [25]. In addition to being dysregulated during various neuroinflammatory disorders, this miRNA was also demonstrated to promote microglia-mediated neuroinflammation by modulating the suppressor of cytokine signaling 1 (SOCS-1) protein, suggesting that the inhibition of miR-155 may be neuroprotective in the context of chronic inflammation [14]. Therefore, confirming the relationship between head trauma and miR-155 regulation could offer a potential biomarker of neuroinflammation and a therapeutic target for treating athletes suffering from chronic illness.

This study was limited in its specificity of data related to the risk of developing diseases these fighters may experience. Individualized data concerning the time of the last professional fight or the most recent concussion is worth considering in determining the timeframe of epigenetic responses to RHI experienced in professional combat. Moreover, the severity and frequency of RHI, factors likely relevant to the neurodegeneration observed in CTE, are challenging to assess. Fighters may underrepresent the number of undiagnosed concussions they have experienced, considering concussions synonymous with losing consciousness or other severe TBI symptoms. mTBI may be difficult for these fighters to self-assess; furthermore, they may be unwilling to disclose signs and symptoms of mTBI for fear of being held back from practice or competition. The predominant fighting style is also worthy of consideration: fighters with more striking experience may be at higher risk of suffering TBI than those who prefer grappling styles, which typically involve more wrestling maneuvers rather than strikes to the head. It remains unclear how body composition could affect the expression of miR-155. A 2021 study suggests that miR-155 mediates obesity-associated β cell dysregulation [26]. While the two groups in this study had significantly different lean masses, there was no difference in body fat (Table 1). Future research can confirm the relationship between miR-155 overexpression and RHI and the potential of this miRNA as a biomarker for individuals at risk for CTE.

Furthermore, work is underway to link the observed up-regulation of miR-155 with other serum-based biomarkers of neurodegeneration, inflammation, and RHI-caused trauma [27]. The expression of other miRNAs (e.g., those found in Table 2) in athletes at various points in their careers or after retirement is also being assessed. These analyses, focused on a more longitudinal approach, may provide the basis to calculate odds ratios based on the medical diagnosis of either the occurrence of concussions or the observation of disease symptoms. The results of this study serve as complimentary findings to this growing body of research.

## Conclusions

This study demonstrated overexpression of miR-155 in a group of professional MMA fighters; furthermore, the miRNA PCR arrays estimated group differences using pooled samples for 27 other miRNAs, which may be significantly altered with participation in professional MMA competition. While these fighters may experience a protective effect from the intense exercise and nutrition regiment they adhere to, accounts continually appear of older retired athletes suffering negative neurobehavioral consequences likely related to repetitive mTBI experienced throughout their careers. Epigenetic research continues to identify sensitive biomarkers involved in disease processes which may aid healthcare professionals in developing treatment plans to maintain the health and well-being of these athletes.
